# Congenital factor XIII deficiency caused by F13A1 gene mutations presenting with intracranial hemorrhage: a case report

**DOI:** 10.3389/fped.2025.1732065

**Published:** 2025-12-19

**Authors:** Hao Wang, Ruotong Yang, Jianchang Li

**Affiliations:** 1 Pediatric Hematology and Endocrinology, Binzhou Medical University Hospital, Binzhou, Shandong, China; 2 Neonatal Intensive Care Unit, Binzhou Medical University Hospital, Binzhou, Shandong, China

**Keywords:** factor XIII deficiency, intracranial hemorrhage, F13A1 gene, infant, genetic diagnosis

## Abstract

This case report describes a male infant with congenital Factor XIII deficiency who presented with severe intracranial hemorrhage. The late preterm infant (36^+4^ weeks) exhibited early signs of bleeding, including a hematoma at an injection site and umbilical stump bleeding. At two months of age, he experienced a spontaneous, grade IV intracranial hemorrhage complicated by hydrocephalus. Notably, routine coagulation studies were within normal limits. The diagnosis was confirmed by genetic testing, which identified compound heterozygous mutations in the F13A1 gene. Management involved external ventricular drainage and regular fresh frozen plasma transfusions as replacement therapy, resulting in a favorable outcome. This case underscores that congenital FXIII deficiency should be considered in the differential diagnosis for infants presenting with unexplained perinatal bleeding or intracranial hemorrhage, especially when standard coagulation screens are normal. Early genetic testing and institution of structured replacement therapy are crucial for preventing life-threatening bleeding and improving long-term prognosis.

## Introduction

Congenital Factor XIII (FXIII) deficiency is a rare autosomal recessive bleeding disorder, predominantly caused by mutations in the F13A1 gene, characterized by impaired fibrin cross-linking during the final stage of coagulation (1). The disease can manifest in the neonatal period as an unexplained bleeding tendency, with delayed umbilical stump bleeding being a classic early sign. Furthermore, affected infants are at risk for life-threatening spontaneous bleeding events, such as intracranial hemorrhage (2, 3). This paper reports a case of spontaneous intracranial hemorrhage secondary to congenital factor XIII deficiency. It includes a literature review to enhance the clinical understanding of this condition and summarize its genetic basis, clinical features, diagnostic approaches, and management strategies.

## Case presentation

A male infant, G2P2, was delivered via cesarean section at a gestational age of 36 weeks and 4 days due to prematurity. Immediately after birth, he received an intramuscular vitamin K injection, which was followed by a persistent soft tissue hematoma at the injection site. On the 8th day of life, he was hospitalized due to umbilical stump bleeding and was discharged after improvement with supportive care.

**Figure 1:**
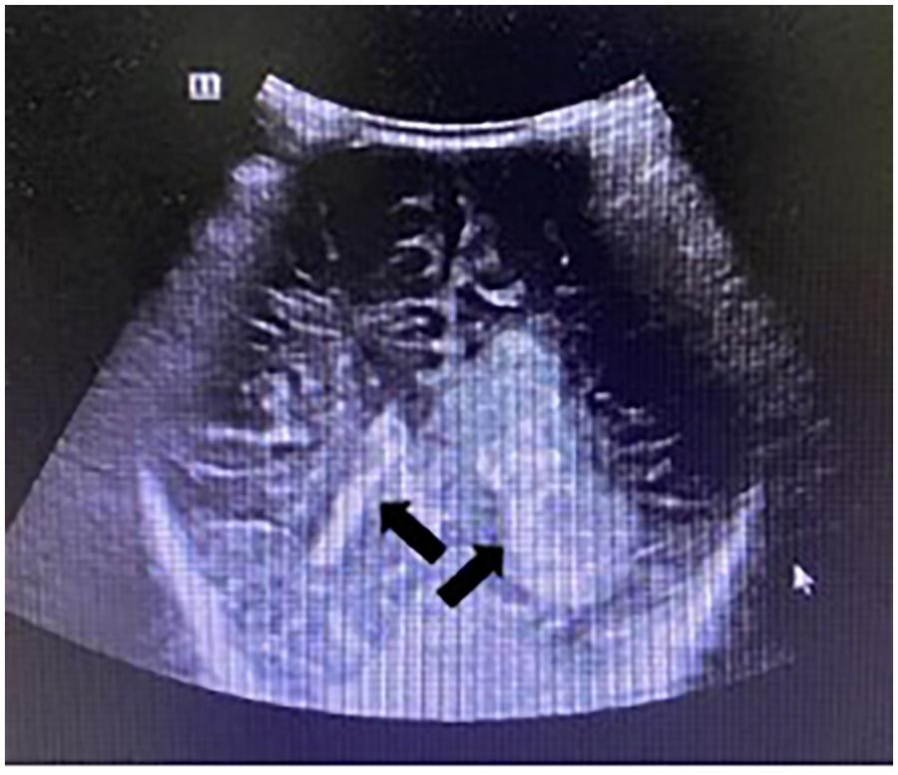
Cranial ultrasonography. Imaging findings revealed a patchy hyperechoic area in the left lateral ventricle and posterolateral region.

At two months of age, he presented to our outpatient department with a 10-hour history of recurrent vomiting. Laboratory tests revealed: red blood cells 3.5 × 10^12^/L, hemoglobin 97 g/L, hematocrit 31%, and platelets 215 × 10^9^/L. Cranial ultrasonography showed a patchy hyperechoic area in the left lateral ventricle and posterior lateral region, suggestive of Grade IV intracranial hemorrhage, along with widening of the anterior horns of the lateral ventricles (Figure 1). Ultrasounds of the hepatobiliary system, pancreas, spleen, kidneys, and gastrointestinal tract were unremarkable. The infant was subsequently admitted to the NICU with a diagnosis of intracranial hemorrhage.

**Figure 2:**
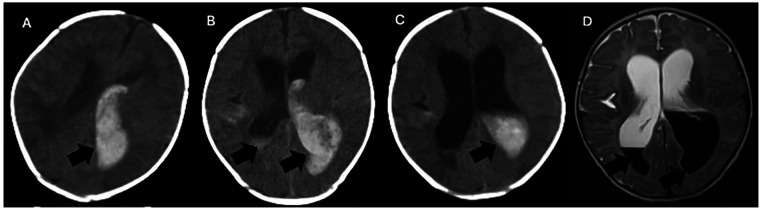
Head CT and MRI. **(A–C)** Serial non-contrast computed tomography (CT) scans of the brain. **(A)** Initial CT: A patchy hyperdensity, suggestive of acute hemorrhage, is seen in the left temporo-occipital lobe and bilateral lateral ventricles. **(B)** Follow-up CT: The intraventricular hyperdensity shows an interval increase in volume and extent compared to **(A)**. **(C)** Subsequent CT: The previously noted hyperdensity demonstrates an interval decrease, consistent with partial resolution of the hemorrhage. **(D)** Corresponding brain magnetic resonance imaging (MRI). A focal lesion within the bilateral lateral ventricles exhibits short T1 and long T2 signal intensities. On the T2-FLAIR sequence, the lesion is hypointense, a signal characteristic consistent with residual methemoglobin.

Upon admission, vital signs were stable. Physical examination revealed a bulging anterior fontanelle measuring approximately 3.0 cm × 3.0 cm; the remainder of the physical examination was unremarkable. Blood gas analysis was essentially normal. A repeat complete blood count indicated worsening anemia (hemoglobin 80 g/L). Cranial CT demonstrated left temporo-occipital lobe hemorrhage with intraventricular and subarachnoid extension (Figure 2). The therapeutic regimen comprised chloral hydrate for sedation, vitamin K1 and etamsylate for hemostasis, albumin combined with furosemide to reduce intracranial pressure, and intravenous nutritional support. A neurosurgery consultation was obtained. Coagulation studies showed slightly decreased levels of some coagulation factors (Tables 1, 2), prompting the transfusion of 75 mL of fresh frozen plasma (FFP). A follow-up CT scan that night revealed increased intraventricular and subarachnoid hemorrhage (Figure 2), and a progressive drop in hemoglobin to 68 g/L necessitated a transfusion of 75 mL of leukocyte-reduced suspended red blood cells.

**Table 1:** Coagulation factor assays.

Parameter		Result	Reference range
Endogenous	Factor II	69.5↓	70–120
	Factor V	86.2	70–120
	Factor VII	83.9	70–120
	Factor X	70.0	70–120
Exogenous	Factor VIII	118.4	70–120
	Factor IX	41.4↓	70–120
	Factor XI	66.3↓	70–120
	Factor XII	68.9↓	70–120

**Table 2:** Coagulation panel with D-dimer.

Parameter	Result	Reference range	Unit
PT	11.9	10–14	S
PT-INR	0.98	0.80–1.50	
PT%	95.1	70–120	%
APTT	37.2↑	23–35	S
TT	19.2	14–21	S
Fib	1.7↓	2–4	g/L
D-Dimer	0.40	0–0.5	mg/L FEU
PT-Ref	12.50	—	S
APTT-R	28.50	—	S

On the second day, the infant exhibited occasional fluctuations in oxygen saturation while on low-flow oxygen, though levels could be maintained within the normal range. His general responsiveness was fair, his skin color was pale, and the anterior fontanelle remained tense. Pupillary light reflexes were slightly sluggish, possibly related to sedative medication. Further consultations with neurosurgery and hematology were sought. Thromboelastography and genetic testing were arranged (Table 3). A repeat blood count showed a hemoglobin level of 96 g/L, leading to a second transfusion of 80 mL of FFP.

**Table 3:** Activated clotting thromboelastography.

Parameter	Result	Reference range	Unit	Parameter	Result	Reference range	Unit
R	6.4	5–10	min	TPI	35.2	5–90	/sec
K	2.2	1–3	min	TMA	26.8	—	Min
Angle	60.2	53–72	deg	E	158.2	92–218	d/sc
MA	61.3	50–70	mm	SP	5.7	—	min
G	7,911.0	4,500–11,000	d/sc	LTE	250.0	—	min
EPL	1.9	0–15	%	PMA	0.0	—	
LY30	1.9	0–8	%	A30	57.5	—	mm
A	51.4	—	mm	CL30	93.9	92–100	%
CI	−0.7	−3–3		CLT	59.9	—	min

R, reaction time; K, kinetics time; Angle, maximum angle/angle (*α*); MA, maximum amplitude; G, clot mechanical strength/firmness; EPL, predicted clot lysis rate; LY30, lysis at 30 min; A, amplitude; CI, coagulation index; TPI, platelet dynamics index; TMA, time to maximum amplitude; E, clot elasticity constant; SP, split point time; LTE, estimated time to total lysis; PMA, projected maximum amplitude; A30, amplitude at 30 min; CL30, clot lysis residual at 30 min.

On day three, the infant's condition remained stable, with no significant changes. Repeat cranial CT (Figure 2), MRI (Figure 2), vascular imaging, and electroencephalography were performed. An urgent neurosurgical consultation concluded that hydrocephalus had developed, and an external ventricular drain was recommended. The family requested transfer to a tertiary care center. There, the infant underwent placement of a left external ventricular drain and a right-sided Ommaya reservoir. Postoperatively, he received intermittent coagulation factor supplementation and Ommaya reservoir taps, leading to clinical improvement and subsequent discharge.

Subsequent genetic testing confirmed the diagnosis of coagulation factor XIII-A subunit deficiency, revealing two pathogenic variants in the F13A1 gene. Currently, the infant receives regular FFP transfusions every 4–6 weeks as factor replacement therapy and maintains a favorable prognosis.

## Discussion

Coagulation factors are core protein components essential for maintaining normal hemostasis. Among them, coagulation factor XIII (FXIII), also known as the fibrin-stabilizing factor, serves as a critical enzyme, the final executor of the coagulation cascade (4). Under physiological conditions, thrombin activates it to FXIIIa, which then acts like a “molecular stitch”, catalyzing the formation of covalent cross-links (*ε*-(*γ*-glutamyl)lysine bonds) between the *γ*-chains and *α*-chains of fibrin monomers. This process transforms the initial fibrin mesh into a stable, structurally dense clot with high mechanical strength, effectively sealing damaged blood vessels (5–8). The recurrent bleeding in our patient, despite normal routine coagulation screening, precisely reflects the unique role of FXIII in consolidating hemostasis—its functional defect does not impair the initiation of coagulation but severely compromises clot stability.

Clinically, FXIII deficiency is categorized into hereditary and acquired forms (Table 4). The hereditary form follows an autosomal recessive inheritance pattern, with a global estimated incidence of 1 in 2–3 million. Consanguinity is associated with a significantly elevated risk. According to the International Society on Thrombosis and Hemostasis (ISTH) algorithm, hereditary FXIII deficiency is categorized into three groups: Type I (FXIII-A deficiency), Type II (FXIII-A deficiency), and FXIII-B deficiency (9). The Type II classification is now considered largely obsolete. To date, 199 different mutations in the F13A1 gene (encoding the A subunit) and 20 mutations in the F13B gene (encoding the B subunit) have been identified in patients with congenital FXIII deficiency (10). Studies demonstrate that while some FXIII-B mutations cause aberrant protein retention within the endoplasmic reticulum and consequent impaired secretion (11, 12), most FXIII-A mutations trigger rapid degradation of the abnormal protein by intracellular proteasomes (13). Acquired FXIII deficiency can be secondary to hepatic or renal dysfunction, inflammatory bowel disease, and myeloid leukemia (14, 15). According to the seminal work by François Duckert, umbilical cord bleeding is the most frequent clinical manifestation of congenital FXIII deficiency, occurring in up to 87% of cases. He also reported intracranial hemorrhage in approximately 25% of patients (16). To this day, umbilical cord bleeding remains the most characteristic hemorrhagic manifestation, highly suggestive of FXIII deficiency (17, 18). Beyond Duckert's work, two large-scale studies involving patients with congenital FXIII deficiency have provided crucial data. Studies in 2003 and 2014, with cohorts of 93 and 190 patients, revealed comparable clinical spectra (17, 19, 20). Conversely, a European study by Ivaskevicius et al. found subcutaneous hemorrhage to be the most common clinical sign, followed by delayed umbilical cord bleeding and muscle hematomas (21). Heterozygous carriers of FXIII deficiency typically have factor activity levels between 50% and 70% and are mostly asymptomatic; however, some studies report that severe bleeding events can occur even in these individuals (22–24). Our patient's clinical course—presenting initially with an injection site hematoma and umbilical stump bleeding, followed later by spontaneous intracranial hemorrhage—aligns perfectly with the typical progression of hereditary FXIII deficiency (25–28). This underscores the importance of maintaining a high index of suspicion for FXIII deficiency in neonates or infants with unexplained perinatal bleeding, even in the presence of normal routine coagulation parameters.

**Table 4:** Type subtype classification of factor XIII deficiency (29–31).

Type	Subtype	Further classification	Mechanism
Congenital	FXIII-A Deficiency	Type I (CRM-)	Markedly reduced or absent antigen levels and activity of the A subunit
		Type I (CRM+)	Normal or mildly reduced antigen levels of the A subunit, with defects in its catalytic function or binding capacity to subunit B and substrates such as fibrinogen
	FXIII-B Deficiency	—	Reduced antigen levels of the B subunit lead to premature activation of the A subunit, resulting in secondary depletion of A subunit levels
	Acquired Combined FXIII-A and FXIII-B Deficiency	—	The FXIII complex fails to assemble and function properly
Acquired	Immune-mediated	Anti-FXIII-A Antibodies	Binds to the catalytic center of the A subunit, inhibiting its transglutaminase activity
			Impairs the binding between the A and B subunits
			Interferes with the activation of FXIII
			Competitively inhibits fibrinogen
		Anti-FXIII-B antibodies	Binds to the B subunit, preventing the formation of the FXIII complex
			Accelerates the clearance of the A₂B₂ complex or the B subunit alone
	Non-Immune	Impaired synthesis	Conditions associated with severe hepatic dysfunction, e.g., cirrhosis, liver failure
			Disorders affecting bone marrow hematopoiesis, e.g., acute myeloid leukemia, myelodysplastic syndromes, primary myelofibrosis
		Enhanced consumption	Major surgery, severe trauma, or extensive burns
			Leukemia
			Disseminated intravascular coagulation (DIC)

The diagnosis of FXIII deficiency remains challenging. Routine coagulation tests (PT/APTT) are incapable of detecting its activity, leading to its frequent oversight in clinical practice. Unexplained bleeding tendencies coupled with impaired clot stability, potentially indicated by reduced Maximum Clot Firmness on thromboelastography, should raise clinical suspicion for this disorder (32). In regions with well-equipped coagulation laboratories, particularly in countries with a high prevalence of FXIII deficiency, quantitative FXIII activity assays are recommended over the traditional clot solubility test (33). The Scientific and Standardization Committee of the ISTH recommends a standardized diagnostic pathway for precise diagnosis and classification. This algorithm begins with a quantitative functional FXIII activity assay as the first-line test. If activity is decreased, subsequent steps include measuring plasma FXIII-A₂B₂ antigen concentration, followed by separate quantification of FXIII-A and FXIII-B subunit antigens to determine the subtype. For suspected platelet FXIII deficiency, analysis of FXIII activity and FXIII-A antigen in platelet lysate is necessary. Mixing studies and binding assays should be performed to detect potential inhibitory antibodies against FXIII subunits. SDS-PAGE analysis of fibrin cross-linking can provide functional validation. Ultimately, molecular genetic testing to identify causative mutations is the definitive step for confirming the etiology and completing the diagnostic workflow (9, 34–36). Ideally, a comprehensive diagnosis should follow guideline recommendations, integrating clinical symptoms and family history (37). However, access to these specialized assays can be limited in practice. In regions with high rates of consanguinity and specific prevalent mutations, genetic screening for common mutations can serve as a cost-effective diagnostic strategy for congenital FXIII deficiency (38). Genetic sequencing confirmed the diagnosis, revealing two pathogenic variants in the F13A1 gene. This highlights the decisive role of genetic testing in diagnosing rare bleeding disorders, especially when clinical suspicion contradicts initial laboratory screening results.

Regular prophylactic treatment is essential in managing FXIII deficiency. Prophylaxis should be initiated upon diagnosis for patients with FXIII levels below 1 IU/dL. For individuals with levels between 1 and 4 IU/dL, who remain at risk for moderate to severe bleeding episodes, prophylaxis is also strongly recommended (39). FXIII replacement therapy is the mainstay for preventing and controlling bleeding. The goal is to raise FXIII levels above the hemostatic threshold, estimated to be between 0.5% and 5%, while maintaining plasma levels between 3% and 10% is generally effective for preventing spontaneous bleeds (40). Acute major bleeding episodes may necessitate higher FXIII trough levels (41–43). Owing to FXIII's relatively long half-life of approximately 5–11 days, common regimens involve transfusions of fresh frozen plasma at 10 mL/kg or cryoprecipitate at 1 bag/10 kg every 4–6 weeks to maintain hemostatic levels (44, 45). However, these blood-derived products carry inherent risks, including imprecise dosing, allergic reactions, and potential transmission of blood-borne pathogens. Regular plasma transfusion, as used in our patient, is a suboptimal therapy. Plasma-derived FXIII concentrate has been available since 1993 and is now considered the first-line international standard for prophylaxis. Dosing typically ranges from 10 to 26 IU/kg every 4–6 weeks, with a regimen of 40 IU/kg every 4 weeks shown to prevent bleeding episodes completely (43). Furthermore, recombinant FXIII-A subunit (rFXIII-A), approved by the US FDA in 2013, eliminates the risks associated with plasma-derived products. A monthly dose of 35 IU/kg maintains plasma FXIII activity above 1% throughout the dosing interval, offering a safer and more precise option for long-term prophylaxis (46, 47). Unfortunately, the accessibility of these specific concentrates, particularly the recombinant form, remains limited in many areas, highlighting the ongoing challenges in securing optimal treatments for rare diseases.

## Conclusion

Hereditary Factor XIII deficiency is a rare disorder characterized by a heterogeneous clinical presentation and typically normal routine coagulation screening tests, posing significant diagnostic challenges and a high risk of being overlooked or misdiagnosed. Insights from this case underscore the critical importance of including FXIII deficiency in the primary differential diagnosis when encountering unexplained bleeding, particularly neonatal umbilical hemorrhage or spontaneous intracranial hemorrhage. Definitive diagnosis relies on specific factor activity assays and genetic analysis. Maintaining a high index of clinical suspicion, promoting access to precise diagnostic tools, and fostering multidisciplinary collaboration are paramount for enabling early intervention. Genetic counseling and prenatal diagnosis should be provided to affected families. For patients with severe deficiency, the timely initiation of regular, long-term prophylactic replacement therapy is essential to prevent life-threatening hemorrhagic events and improve overall prognosis.

## Data Availability

The original contributions presented in the study are included in the article/Supplementary Material, further inquiries can be directed to the corresponding author.
